# Stellungnahme der Deutschen Gesellschaft für Rheumatologie e. V. (DGRh) zur Anwendung der Videosprechstunde in der Rheumatologie

**DOI:** 10.1007/s00393-020-00932-x

**Published:** 2020-11-17

**Authors:** P. Aries, M. Welcker, J. Callhoff, G. Chehab, M. Krusche, M. Schneider, C. Specker, J. G. Richter

**Affiliations:** 1 Rheumatologie im Struenseehaus, Hamburg, Deutschland; 2 Geschäftsführung, MVZ für Rheumatologie Dr. Martin Welcker GmbH & RheumaDatenRhePort (RhaDaR), Planegg, Deutschland; 3 Programmbereich Epidemiologie, Deutsches Rheuma-Forschungszentrum (DRFZ), Berlin, Deutschland; 4 Poliklinik und Funktionsbereich Rheumatologie & Hiller-Forschungszentrum Rheumatologie, Medizinische Fakultät, Universitätsklinikum Düsseldorf, Heinrich-Heine-Universität Düsseldorf, Moorenstr. 5, 40225 Düsseldorf, Deutschland; 5 Medizinische Klinik mit Schwerpunkt Rheumatologie und Klinische Immunologie, Charité Universitätsmedizin, Berlin, Deutschland; 6 Klinik für Rheumatologie & Klinische Immunologie, Evangelisches Krankenhaus, Kliniken Essen-Mitte, Essen, Deutschland

**Keywords:** Medizinische Versorgung, COVID-19-Pandemie, Rheumatologische Versorgung, Telemedizin, Telekonsultation, Medical care, COVID-19 pandemic, Rheumatological care, Telemedicine, Teleconsultation

## Abstract

Die Durchführung von Videosprechstunden wird seit mehreren Jahren als ergänzende Form der medizinischen Versorgung in Ergänzung zu einem persönlichen Arzt-Patienten-Kontakt angesehen und teilweise auch gefördert. Die COVID-19-Pandemie hat der Nutzung von Videosprechstunden ungeahnte Aktualität und Aufmerksamkeit verschafft. Die Kassenärztliche Bundesvereinigung beschloss Sonderregelungen im Rahmen der COVID-19-Pandemie, die bisherige Hindernisse für den Einsatz von Telemedizin und Videosprechstunden (und auch teilweise der herkömmlichen Telefonie) reduziert. Die vorliegende Stellungnahme der DGRh zum Einsatz von Videosprechstunden soll einen Überblick darüber geben, in welcher Form und mit welchen Limitierungen die Videosprechstunde in der Rheumatologie in Deutschland anwendbar ist. Die Stellungnahme skizziert einen Ausblick, wie die Videosprechstunde welche Funktionen zukünftig in der rheumatologischen Versorgung übernehmen kann.

## Abstract (en)

For several years video consultations have been regarded as a new form of medical healthcare infrastructure, in addition to personal doctor-patient contacts and have also been partly promoted. The COVID-19 pandemic brought unexpected topicality and attention to the use of video consultations. The National Association of Statutory Health Insurance Physicians (Kassenärztliche Bundesvereinigung) decided on special regulations in the context of the COVID-19 pandemic, which reduce previous obstacles to the use of telemedicine and video consultations (and also partly of conventional telephony). The present statement of the German Society of Rheumatology (DGRh) on the use of video consultations is intended to give an overview of in which form and with which limitations video consultations can be used in rheumatology in Germany. It sketches an outlook on how video consultations can undertake which functions in rheumatological care in the future.

## 1 Einleitung

Die Durchführung von Videosprechstunden wird seit mehreren Jahren als Teil moderner Versorgungsstrukturen angesehen und gefördert. Die Akzeptanz bei Ärzten war aus unterschiedlichen Gründen bis vor Kurzem tatsächlich aber gering [1].

Durch die COVID-19-Pandemie hat sich im Rahmen des Infektionsschutzgesetzes sowie der verordneten Kontakt- und Mobilitätsbeschränkungen die digitale Kommunikation in der medizinischen Versorgung nahezu disruptiv entwickelt. Nach Angaben der KBV stieg die Zahl der Ärzte, die sich für eine Videosprechstunde haben registrieren lassen, in kürzester Zeit auf ca. 25.000 [2]. Berücksichtigt man, dass insgesamt ca. 149.000 Ärzte an der vertragsärztlichen Versorgung in Deutschland teilnehmen, würde rechnerisch knapp ein Fünftel aller Ärzte diese neue Konsultationsform ihren Patienten anbieten [3]. Neben den Hausärzten gehören die 12.400 Fachinternisten wahrscheinlich zu der Gruppe von Ärzten, für die aufgrund eines hohen Beratungsaufwandes der Einsatz der Videosprechstunde, gerade auch in der aktuellen Situation der Pandemie, naheliegend ist. Auch wenn Zahlen für die Rheumatologie aktuell noch fehlen, ist davon auszugehen, dass auch rheumatologische Einrichtungen in Deutschland eine digitale Form der Patientenbetreuung durchführen oder in Zukunft planen durchzuführen.

Hieraus leitet sich aus Sicht der DGRh die Notwendigkeit ab, neben den grundsätzlichen bereits gesetzten rechtlichen und abrechnungstechnischen Rahmenbedingungen den Stellenwert der Videosprechstunde in der Rheumatologie zu analysieren bzw. ihre künftigen Einsatzmöglichkeiten zu diskutieren. Dies soll auch dazu dienen, notwendige Evaluationen dieser in der Rheumatologie bislang wenig genutzten Versorgungsstruktur zu unterstützen.

## 2 Literaturübersicht

In der bis dato einzigen kontrollierten, kanadischen Studie zur Anwendung der Videosprechstunde in der Rheumatologie fand sich bei bekannten Patienten kein qualitativer Unterschied (bezogen auf DAS28-CRP, RADAI, mHAQ und EQ5D) zwischen der Videokonferenz und der persönlichen Vorstellung bei Patienten mit etablierter rheumatoider Arthritis. Insbesondere die Vorteile aufseiten der Patienten wurden als wesentlicher Nutzen hervorgehoben, um die Kontinuität der rheumatologischen Versorgung aufrechterhalten zu können. Allerdings gab es eine deutlich erhöhte Drop-out-Rate im Studienarm mit Versorgung per Videosprechstunde (43 % Drop-out bei Videosprechstunde, 26 % bei persönlicher Vorstellung) [4].

Ein systematisches Review aus dem Jahr 2017 weist nach der Auswertung von 19 Beobachtungsstudien darauf hin, dass die Effektivität der Telerheumatologie unterschiedlich beurteilt wird, es aber Hinweise für Kosteneffektivität gibt. Da die Wirksamkeit der Telemedizin in der Rheumatologie möglichweise je nach Krankheit, Behandlungsphase und den verwendeten telemedizinischen Methoden variieren könnte, wird die weitere wissenschaftliche Begleitung solcher Projekte gefordert [5].

Zur Nutzung der Videosprechstunde bei Rheumatologen und deren Hemmnissen liegen bislang nur wenige Daten vor. In einer Umfrage unter den Hausärzten und Rheumatologen des Landes Brandenburg (2395 Ärzte, Rücklaufquote 20,8 %), die sich mit verschiedenen Aspekten der Telemedizin einschließlich der Videosprechstunde beschäftigte, gab knapp die Hälfte der befragten Ärzte (48,4 %) an, „ungenügende“ oder „mangelhafte“ Kenntnisse der Telemedizin, in deren begriffliche Verortung die Videosprechstunde gehört, zu haben, wobei die „Anschaffung der Technik“ (62,3 %), der „administrative Aufwand“ (62,0 %) sowie die „schwache Vergütung“ (53,4 %) als maßgebliche Hindernisse für deren Einsatz angegeben wurden [6]. Die Durchführung von Kontrollterminen (71,1 %) und das Screening von Patienten (51,1 %) wurden seitens der Teilnehmer als möglicherweise sinnvolle Einsatzszenarien der Telemedizin benannt. Der Nutzung von Telemedizin beim Erstkontakt stimmten hingegen lediglich 24,4 % der Befragten zu.

## 3 Gesetzliche Grundlage

Mit der Verabschiedung des E‑Health-Gesetzes („Gesetz für sichere digitale Kommunikation und Anwendungen im Gesundheitswesen sowie zur Änderung weiterer Gesetze“, gültig ab 29.12.2015) wurde Ende 2015 der Digitalisierung in der Medizin seitens des Gesetzgebers ein neuer rechtlicher Rahmen gesetzt. Es folgten zeitnah Aktualisierungen und Anpassungen in den Jahren 2016 und 2017.

Das E‑Health-Gesetz stellt einen Fahrplan für den Aufbau der sicheren Telematikinfrastruktur und die Einführung medizinischer Anwendungen in der Versorgung dar. Ziel dieses Gesetzes ist es, die Chancen der Digitalisierung für die Gesundheitsversorgung zu nutzen und eine schnelle Einführung medizinischer Anwendungen für die Patientinnen und Patienten zu ermöglichen. Die Organisationen der Selbstverwaltung erhalten darin klare Vorgaben und Fristen, die bei Nichteinhaltung teilweise auch zu Sanktionen führen können [7].

In Folge wurden die Videosprechstunde anfänglich mit der telekonsiliarischen Befundbeurteilung von Röntgenaufnahmen (ab April 2017) und dann die Online-Videosprechstunde (ab Juli 2017) in die vertragsärztliche Versorgung aufgenommen. Das geschah mit dem Ziel, Patienten die Kontaktaufnahme mit dem Arzt, gerade bei Nachsorge- und Kontrollterminen, zu erleichtern [8]. Das Fernbehandlungsverbot wurde allerdings erst im Folgejahr, im Mai 2018 auf dem 121. Deutschen Ärztetag, gelockert bzw. aufgehoben [9]. Die rechtliche Umsetzung der „Fernbehandlung“ wurde dabei in den einzelnen KV-Bezirken durchaus unterschiedlich gehandhabt [10]. So ist bzw. war es bislang notwendig zu beachten, ob der beratene Patient sich zum Zeitpunkt der Videokommunikation auch in einer KV-Region aufhielt, die dieses Vorgehen auch als ärztliche Leistung vorsieht. Im Jahr 2019 wurde die Videosprechstunde auch in die Leistungsagenda der ambulant spezialärztlichen Versorgung aufgenommen [11]. Die Durchführung der Videosprechstunde in den Hochschulambulanzen ist nach Kenntnis der Autoren derzeit nicht einheitlich geregelt.

Die weitere Ausgestaltung der Digitalisierung in der Medizin erfolgte 2019 mit der Verabschiedung des „Digitalen-Versorgung-Gesetzes“ (DVG). Dieses wurde durch die „Digitale Gesundheitsanwendungen Verordnung“ (DiGAV) für die praktische Umsetzung ausgestaltet und am 20.04.2020 im Bundesanzeiger veröffentlicht [12]. Diese Regularien sollen helfen, die Kommunikation der Partner im Gesundheitswesen zu vereinfachen z. B. durch die Einführung von Online-Sprechstunden (diese sollen nach Ausdruck des Gesetzgebers „Alltag“ werden) und auch die Verschreibung sog. Gesundheits-Apps zu fördern [13, 14].

Vor der Durchführung einer Videosprechstunde sind *grundsätzliche* Voraussetzungen zu beachten. Hierzu gehört, dass in jedem Einzelfall zu prüfen ist, ob die *ausschließliche* Fernbehandlung „ärztlich vertretbar“ ist und ob die erforderliche ärztliche Sorgfalt durch die Art und Weise dieser Form der Befunderhebung, Beratung, Behandlung sowie die Dokumentation ausreichend gewahrt ist [15]. Gesetzliche Dokumentationsvorgaben dazu sind bislang allerdings nicht veröffentlicht. Das Ausstellen von elektronischen Rezepten und elektronischen Arbeitsunfähigkeitsbescheinigungen ist in der Videosprechstunde bisher nicht erlaubt. Zudem darf auch die Videosprechstunde nicht aktiv beworben werden (§ 9 Heilmittelwerberecht).

Anfang April 2020 wurden seitens der Kassenärztlichen Bundesvereinigung (KBV) Sonderregelungen im Rahmen der COVID-19-Pandemie beschlossen, welche die Begrenzungen der Telemedizin und Videosprechstunde (aber auch teilweise der Telefonie) reduzierten (u. a. https://www.kbv.de/media/sp/PraxisInfo_Coronavirus_Videosprechstunde.pdf):

- Aufhebung der Fallzahl und Leistungsmengenbegrenzung bis 30.06.2020,
- Ausweitung auf alle Indikationen und Möglichkeit der Durchführung bei bisher nicht in der Praxis bekannten Patienten.

Klare rechtliche Aussagen, welche Krankheitsstadien und/oder bis zu welcher Krankheitsaktivität entzündlich rheumatische Erkrankungen per Videosprechstunde versorgt werden dürfen, wurden bislang noch nicht gemacht. Dies gilt auch für die Versorgung unerwünschter Wirkungen medikamentöser Therapien respektive andere Komplikationen.

Aktuell gehaltene Übersichten zu zertifizierten Videodienstanbietern stehen auf den Webseiten der KBV zur Verfügung (https://www.kbv.de/html/1150_44943.php).

## 4 Vertragsärztliche Voraussetzung (GKV)

Ärztinnen und Ärzte können die Videosprechstunde flexibel in allen Fällen nutzen, in denen sie es für „therapeutisch sinnvoll“ halten. Möglich ist das mittlerweile sowohl bei bekannten als auch bisher unbekannten Patientinnen und Patienten [16].

Vonseiten des Gesetzgebers und der kassenärztlichen Bundesvereinigung wurden Voraussetzungen für Praxen und Videodienstanbieter formuliert, die insbesondere die technische Sicherheit und den Datenschutz unter anderem in der Anlage 31b zum Bundesmantelvertrag-Ärzte regelt (Tab. 1 und 2; [17]). Einen stets aktuell gehaltenen Überblick über die zertifizierten Videodienstanbieter bietet die KBV (https://www.kbv.de/html/videosprechstunde.php). Wichtig ist dabei zu berücksichtigen, dass Ärzte vertragsärztliche Leistungen im Rahmen der Videosprechstunde erst dann abrechnen können, wenn sie ihrer kassenärztlichen Vereinigung zuvor angezeigt haben, einen zertifizierten Videodienstanbieter zu nutzen. Die genaue Angabe, welcher Anbieter genutzt wird, ist dabei nicht zwingend notwendig. In einigen Regionen muss allerdings eine Vertragsbestätigung des zertifizierten Anbieters vorgelegt werden. Praxen und andere ambulante Strukturen sollten sich daher vor Abrechnung der Videosprechstunde bei ihrer zuständigen Kassenärztlichen Vereinigung informieren (https://www.kbv.de/media/sp/Anzeige_Videosprechstunde_KV.pdf).

**Table 1:** 

Technische Voraussetzungen	Anforderung an Praxis
Zertifizierter Videodienstanbieter	Patienteneinwilligung (schriftlich oder zu Beginn jeder Sitzung)
Ende-zu-EndeVerschlüsselung	Raum und Technik für angemessene Kommunikation
Keine Werbung	Gewährleistung der Vertraulichkeit (Aufzeichnungen, z. B. Video, durch Arzt *und* Patient sind nicht erlaubt)
Patientenidentifikation mittels Klarnamen	Anmeldung der Nutzung eines Videodienstanbieters bei der zuständigen KV (eine Nennung des Namens des genutzten Anbieter ist teilweise nicht erforderlich)

**Table 2:** 

Registrierung des Arztes bei einem zertifizierten Videodienstanbieter		
Zuweisung eines Videosprechstundentermins für den Patienten über den Videodienstanbieter oder die Praxis		
Erneute Einwilligungserklärung des Patienten vor der ersten Videosprechstunde anonym über den Videodienstanbieter oder direkt über den Arzt, auch wenn keine Nachweispflicht besteht, empfiehlt sich die erneute Dokumentation durch den Arzt		
In jedem Einzelfall ist zu prüfen, ob die ausschließliche Fernbehandlung „ärztlich vertretbar“ ist und ob die erforderliche ärztliche Sorgfalt durch die Art und Weise der Befunderhebung, Beratung, Behandlung sowie Dokumentation ausreichend gewahrt ist		
Einwahl Patient und Arzt beim Videodienstanbieter, Arzt ruft Patienten im Online-Wartezimmer auf		
Patientenidentifikation	Bekannter Patient	Mündliche Bestätigung durch den Patienten über einen bestehenden Versicherungsschutz und Dokumentation durch den Arzt
	Neuer Patient	Dokumentation der elektronischen Gesundheitskarte (Bezeichnung der Krankenkasse; Name, Vorname und Geburtsdatum des Versicherten; Versichertenart; Postleitzahl des Wohnortes; Krankenversichertennummer)
		Mündliche Bestätigung durch den Patienten über einen bestehenden Versicherungsschutz
Durchführung Videosprechstunde		
Abmeldung von Internetseite nach Videosprechstunde		
Dokumentation der Behandlung im PVS/KIS/Abrechnungssystem		

## 5 Abrechnung

### 5.1 EBM

Die Videosprechstunde ist bei allen Indikationen und auch dann möglich, wenn der Patient unabhängig von der Fachrichtung zuvor nicht bei dem Arzt in Behandlung war („Neuvorstellung“) [18]. Die Abrechnung der Videosprechstunde ist mit der „Pseudo-GOP 88220“ zu kennzeichnen, wenn der Patient in einem Quartal ausschließlich die Videosprechstunde aufgesucht hat. Bisher galt die Regelung, wonach die Anzahl dieser Behandlungsfälle auf 20 % aller Behandlungsfälle des einzelnen Arztes beschränkt war.

Die KBV und die Krankenkassen haben die geltenden Beschränkungen für den Einsatz der Videosprechstunde für das zweite und dritte Quartal 2020 Corona-bedingt aufgehoben (vgl. https://www.kbv.de/html/1150_46724.php). Damit sind sowohl die Fallzahl als auch die Leistungsmenge nicht mehr limitiert (Tab. 3).

**Table 3:** 

GOP		
*13691/2*	Grundpauschale	25 %iger Abschlag, wenn der Patient im Quartal ausschließlich mittels Videosprechstunde betreut wurde
*13700*	Zusatzpauschale für internistische Rheumatologen	Bei Vorliegen der patientenbezogenen Voraussetzungen auch bei reinem Videokontakt abrechenbar
*13701*	Zusatzpauschale rheumatologische Funktionsdiagnostik	Voraussetzungen für die Abrechnung der GOP 13701 entsprechend dem Verständnis der KBV nicht erfüllt Wird von einzelnen KVen unterschiedlich gehandhabt, z. B. KV Hamburg
*01450*	Technik- und Förderzuschlag	Zusätzlich zur Grundpauschale für Kosten des Videosprechstundendienstes Er ist bei jeder einzelnen Videosprechstunde einzugeben. Vor der Pandemie war der Zuschlag pro Quartal auf 205,52 € pro Arzt gedeckelt
*01451*	Anschubfinanzierung	Anschubfinanzierung von 10,00 €, maximal 500 € Fördermöglichkeit für die ersten 2 Jahre, bei mindestens 15 Videosprechstunden im Quartal Die Mengenbegrenzung wurde von der KV in Zeiten der Pandemie für das Quartal 2/2020 ausgesetzt
*01444*	Aufwandsentschädigung Stammdatenerfassung	Aufwand zur Identifizierung eines in der Praxis „unbekannten“ Patienten, extrabudgetär vergütet Patient hält seine elektronische Gesundheitskarte in die Kamera Patient soll mündlich das Bestehen des Versicherungsschutzes bestätigen
*88420*	SARS-CoV-2-Zuschlag	„Alle ärztlichen Leistungen, die aufgrund des begründeten klinischen Verdachts auf eine Infektion oder einer nachgewiesenen Infektion mit dem Coronavirus erforderlich sind, werden seit 1. Februar in voller Höhe bezahlt“ (https://www.kbv.de/html/coronavirus.php#content45248) An allen Tagen einer Behandlung zu dokumentieren Weitere Ziffern werden auch extrabudgetär vergütet

### 5.2 GOÄ

Die Abrechenbarkeit der Videosprechstunde in der privatärztlichen Versorgung ist im Vergleich zu der GKV deutlich eingeschränkter (Tab. 4).

**Table 4:** 

*Ziffer 1*	„Beratung“	
*Ziffer 3*	„Eingehende Beratung“	Bei einer Mindestdauer von 10 min berechnungsfähig, wobei eine „mehr als einmalige Berechnung“ im Behandlungsfall einer besonderen Begründung bedarf
*Ziffer 4*	„Fremdanamnese über einen Kranken und/oder Unterweisung und Führung der Bezugsperson im Zusammenhang mit der Behandlung eines Kranken“	Zu diskutieren, je nach Krankenkasse möglich
*Ziffer 5*	„Symptombezogene Untersuchung“	Formal und auch praktisch abrechenbar, allerdings kritisch zu diskutieren, da eine adäquate „Untersuchung“ (z. B. vollständiger Gelenkstatus) anhand eines Videobildes nicht gewährleistet ist, auch wenn in anderen Fachbereichen wie der Dermatologie die „Blickdiagnose“ in den Alltag integriert ist
*Zuschlag*		
A	Außerhalb der Sprechzeiten	
B	Zwischen 20 und 22 Uhr oder 6 und 8 Uhr	
C	Zwischen 22 und 6 Uhr	
D	Samstag, Sonntag oder Freitag	

## 6 Ausblick

Die Videosprechstunde als Teil der Telemedizin kann als in Zukunft zunehmend relevanter Beitrag zur rheumatologischen Versorgung in Deutschland verstanden werden. Die DGRh ist dabei der Auffassung, dass die Videosprechstunde nur unter bestimmten, qualitätssichernden Voraussetzungen (z. B. begleitende Versorgungsforschung) die bisherige rheumatologische Regelversorgung ergänzen sollte. Aufgrund der qualitativen Einschränkungen (keine direkte körperliche Untersuchung, keine apparative Untersuchung wie Sonographie etc.) kann die Videosprechstunde nicht als vollständiger Ersatz für eine persönliche Betreuung vor Ort verstanden werden. Ausgehend von der aktuellen Pandemiesituation hält die DGRh die Videosprechstunde zum gegenwärtigen Zeitpunkt unter Beachtung oben genannter Einschränkungen und soweit sich keine neue Daten- oder Rechtslage ergibt für eine sinnvolle Ergänzung der rheumatologischen Versorgung, v. a. in den nachfolgend aufgeführten Situationen.

### In Zeiten der Pandemie

1. Zur allgemeinen Prävention von SARS-CoV-2-Infektionen durch Kontaktminimierung und Reduktion der Mobilität
2. Zur Fernbetreuung von:
  - COVID-19-erkrankten Patienten
  - SARS-CoV-2-infizierten Patienten
  - Patienten in angeordneter/freiwilliger Quarantäne
  - Patienten mit erhöhtem Risiko für eine SARS-CoV-2-Infektion
3. Als partiellen Ersatz für regelmäßige Kontrollen zur Therapie- und Krankheitsüberwachung vor Ort

### Außerhalb von Pandemiezeiten

Außerhalb der Pandemie und perspektivisch in Zukunft bietet die Videosprechstunde aus der Sicht der DGRh eine Chance zur Optimierung der rheumatologischen Versorgung, auch wenn es noch gilt, für die nachfolgend genannten Punkte Evidenz zu schaffen:

1. zur Erweiterung des Spektrums der rheumatologischen Versorgung
  - mit Schließung regionaler Versorgungslücken,
  - mit Erweiterung der rheumatologischen Sprechstundentermine (u. a. außerhalb der üblichen Praxiszeiten),
  - mit Ermöglichung einer Screeningsprechstunde bei Verdacht auf eine entzündlich rheumatische Erkrankung zur weiteren Terminpriorisierung;
2. zur Umsetzung/Optimierung moderner Therapiekonzepte wie
  - „shared decision“,
  - „tight control“ und
  - „treat to target“.

Für die meisten der oben genannten Punkte gilt, dass der niedrigschwellige Zugang zur rheumatologischen Versorgung insbesondere in besonderen Situationen wie der Pandemie zu einer Optimierung der rheumatologischen Versorgung führen kann, indem Patienten eine rheumatologische Versorgung erfahren können, denen in den genannten besonderen Situationen der Zugang zu den rheumatologischen Praxen und Ambulanzen ansonsten verwehrt geblieben wäre.

Bei Einsatz der Videosprechstunde ist jedoch zu berücksichtigen, dass nicht alle entzündlich rheumatischen Erkrankungen und jegliche Krankheitsstadien in gleicher Form für eine Betreuung mittels Videosprechstunde geeignet sind. Insbesondere bei Neupatienten, bei Patienten mit unzureichender Kontrolle der Krankheitsaktivität bzw. unzureichender Verträglichkeit der Therapie sowie bei Systemerkrankungen muss noch geprüft werden, ob eine Videosprechstunde geeignet ist, Präsenztermine zu ersetzen. Dagegen hält die DGRh eine Videosprechstunde bei bekannten Patienten, mit guter Kontrolle der Krankheitsaktivität und ausreichender Verträglichkeit der Therapie für eher geeignet, wenngleich auch diese Aussage noch evaluiert werden muss. Eine langfristige, ausschließlich Videosprechstunden gestützte Betreuung wird unter Berücksichtigung aktueller Qualitätsstandards und Leitlinien in der Rheumatologie bislang und auf absehbare Zeit als nicht möglich eingestuft, da eine Videosprechstunde die direkte, physische Arzt-Patient-Interaktion nicht vollständig ersetzen kann. Insbesondere das Problem der „sozialen Distanz“ ist bei der Betreuung von Patienten mit chronischen Erkrankungen nicht von der Hand zu weisen. So spielt die geringe räumliche Distanz eine zentrale Rolle bei der Bildung von Vertrauen, Erleben von Empathie und nicht zuletzt auch der Aufrechterhaltung der Aufmerksamkeit im Gespräch. Insbesondere Patientengruppen mit besonderem Betreuungsbedarf (z. B. ältere Patienten, sozial schwache Patienten) sollten bei der Evaluation von Videosprechstunde spezielle Aufmerksamkeit erfahren. Auch wenn Aspekte wie die „soziale Distanz“ und der z. B. ökonomisch begründet fehlende Zugang zu modernen Medien zu beachten sind, kann die Videosprechstunde auch zu einer besseren rheumatologischen Versorgung führen, da sie – spätestens, wenn sie in die Routineversorgung Eingang gefunden hat – eine häufigere und niederschwelligere Kommunikation der Patienten mit rheumatologischen Fachkräften ermöglichen würde.

Während die Videosprechstunde so v. a. eine quantitative Verbesserung der Versorgung ermöglichen könnte, ist darauf hinzuweisen, dass sie eindeutig mit qualitativen Einschränkungen gegenüber einer persönlichen Visite einhergeht. Für die adäquate Betreuung der Patienten mit rheumatologischen Erkrankungen notwendige differenzialdiagnostische Verfahren – wie klinische, technische und laborchemische Untersuchungen – fehlen bei dem alleinigen Einsatz der Videosprechstunde komplett. Dieses könnte im ungünstigen Fall zu Fehldiagnosen oder inadäquaten therapeutischen Entscheidungen führen – ein Problem, das allerdings bei einer schon seit vielen Jahrzehnten erfolgenden, abrechenbaren und nicht mit Restriktionen des Datenschutzes oder einer fehlenden Evaluation „belasteten“ telefonischen Beratung in noch größerem Umfang anzunehmen ist.

Nicht zuletzt ist auch die Ressource „Zeit“ in Bezug auf die Videosprechstunde von wesentlicher Bedeutung. Es bliebe zu prüfen, wie viel Zeitressourcen eine virtuelle Visite gegenüber einer durch sie ersetzten Präsenzvisite beansprucht. Anzunehmen sind Vorteile aufseiten der Patienten, die sich im Falle einer Videosprechstunde die Zeit der An- und Abfahrt zu Praxis bzw. Klinik und ggf. auch noch die Wartezeit im Wartezimmer ersparen. Vergleichbare Vorteile für die Ärzte ergeben sich dagegen nicht zwangsläufig, sodass wahrscheinlich von keiner wesentlichen Zeitersparnis auf ärztlicher Seite auszugehen ist. Der zusätzliche technische und organisatorische Aufwand für die Durchführung digitaler Sprechstunden ist auf ärztlicher und Patientenseite nicht unerheblich und wird bei der Beurteilung des Aufwandes für eine Videosprechstunde häufig unterschätzt. Bei zunehmend routinemäßiger Nutzung solcher neuer Technologien wird sich dieser Aufwand aber möglicherweise in Zukunft reduzieren.

Eine Studie aus Indien berichtet kürzlich, dass die Umstellung auf Telekonsultation in COVID-19-Zeiten durchführbar war und von den Patienten akzeptiert wurde [19]. Die COVID-19-Pandemie hat uns anschaulich die Grenzen und Risiken der aktuell weitgehend analogen Patientenversorgung und die Bedeutung und Chancen der Digitalisierung aufgezeigt. Um aktuell und in Zukunft die Möglichkeiten der Digitalisierung und insbesondere der Videosprechstunde adäquat nutzen zu können, bedarf es eines verantwortungsvollen, longitudinal zu evaluierenden Einsatzes dieser modernen Versorgungsform. Auch wenn die technischen Voraussetzungen im Rahmen der Pandemie erfreulicher schnell von vielen umgesetzt werden konnten, hängt der zukünftige Einsatz dieser Methode maßgeblich von zusätzlichen organisatorischen, datentechnischen und letztlich auch finanziellen Faktoren ab.

Neben der Kompetenzvermittlung und der organisatorischen Unterstützung der aktuell an der Versorgung teilnehmenden Rheumatologen und Einrichtungen wäre die frühzeitige Heranführung der zukünftigen Ärztegeneration an die digitale Medizin und ihre telemedizinischen Versorgungsformen bereits an den Universitäten wünschenswert.

In der Vergangenheit hatte sich gezeigt, dass die Höhe der Vergütung, aber auch die Transparenz und Dauerhaftigkeit der Vergütungsstrukturen entscheidende Faktoren sind, ob sich Prozesse wie die Videosprechstunde langfristig in der rheumatologischen Versorgung etablieren können. Hier sind die Entwicklungen und Vorgaben der KBV abzuwarten. Solange der Mehraufwand, insbesondere gegenüber einem Telefonat, lediglich nur einen geringen finanziellen Vorteil hat und dieser möglicherweise durch Mengenbegrenzung limitiert und zeitlich abhängig ist, werden die Leistungserbringer die Videosprechstunde nicht breit einsetzen. Solche Hemmnisse in der Umsetzung moderner telemedizinischer Anwendungen müssen erwähnt werden, auch wenn grundsätzlich Aspekte einer verbesserten oder auch nur zeitlich optimierten Versorgung im Vordergrund stehen.

Aus der Sicht der DGRh ist die wissenschaftliche Evaluation der Videosprechstunde wesentliche Voraussetzung für einen adäquaten Einsatz und auch für eine adäquate Vergütung in Zukunft. Nur der wissenschaftliche Nachweis, dass die quantitative Versorgung nachhaltig verbessert wird, ohne erhebliche Einbußen bei der qualitativen Versorgung in Kauf nehmen zu müssen, wird zur langfristigen Etablierung der Videosprechstunde in der Rheumatologie führen können – auch ohne dass es hierzu einer umwälzenden Pandemie bedarf.

## References

[1] Mühlensiepen F, Machbarkeitsstudie MW Implementierung eines telemedizinischen Versorgungskonzeptes in die Rheumatologie im Land Brandenburg (TeleRheumaBB) – Zwischenergebnisse. https://www.egms.de/static/en/meetings/dgrh2018/18dgrh051.shtml;. Zugegriffen: 25. Mai 2020

[2] https://www.kbv.de/html/1150_45755.php;. Zugegriffen: 17. Apr. 2020

[3] KBV Statistische Informationen aus dem Bundesarztregister. https://gesundheitsdaten.kbv.de/cms/html/16393.php;. Zugegriffen: 25. Mai 2020

[4] Taylor-Gjevre R, Nair B, Bath B (2018). Addressing rural and remote access disparities for patients with inflammatory arthritis through video-conferencing and innovative inter-professional care models. Musculoskelet Care.

[5] McDougall JA, Ferucci ED, Glover J, Fraenkel L (2017). Telerheumatology: a systematic review. Arthritis Care Res.

[6] Mühlensiepen F, Marquardt W, Welcker M Machbarkeitsstudie: Implementierung eines telemedizinischen Versorgungskonzeptes in die Rheumatologie im Land Brandenburg (TeleRheumaBB) – Zwischenergebnisse der Fragebogenerhebung. https://www.egms.de/static/en/meetings/dgrh2019/19dgrh116.shtml;. Zugegriffen: 25. Mai 2020

[7] https://www.bundesgesundheitsministerium.de/service/begriffe-von-a-z/e/e-health-gesetz.html;. Zugegriffen: 25. Mai 2020

[8] BMG E‑Health-Gesetz verabschiedet. https://www.bundesgesundheitsministerium.de/ministerium/meldungen/2015/e-health.html;. Zugegriffen: 25. Mai 2020

[9] https://www.kbv.de/html/videosprechstunde.php;. Zugegriffen: 25. Mai 2020

[10] Hillienhof A (2018). Berufsordnung: Ja und vorerst Nein zur ausschließlichen Fernbehandlung. Dtsch Arztebl.

[11] https://www.g-ba.de/downloads/40-268-5673/2019-03-22_ASV-RL_Aktualisierung-Appendizes_TrG.pdf;. Zugegriffen: 12. Aug. 2020

[12] https://www.bgbl.de/xaver/bgbl/start.xav?startbk=Bundesanzeiger_BGBl&start=//*%5B@attr_id=%27bgbl120s0768.pdf%27%5D#__bgbl__%2F%2F*%5B%40attr_id%3D%27bgbl120s0768.pdf%27%5D__1590174001180. Zugegriffen: 25. Mai 2020

[13] https://www.bundesgesundheitsministerium.de/digitale-versorgung-gesetz.html;. Zugegriffen: 25. Mai 2020

[14] Digitale-Versorgung-Gesetz. https://www.bgbl.de/xaver/bgbl/start.xav?startbk=Bundesanzeiger_BGBl&jumpTo=bgbl119s2562.pdf#__bgbl__%2F%2F*%5B%40attr_id%3D%27bgbl119s2562.pdf%27%5D__1596531630200. Zugegriffen: 4. Aug. 2020

[15] https://www.telemedbw.de/FAQ-Haftung/haftung-bei-verstoss-gegen-aerztliche-sorgfaltspflicht. Zugegriffen: 7. Aug. 2020

[16] https://www.kbv.de/html/1150_44943.php;. Zugegriffen: 25. Mai 2020

[17] Bundesmantelvertrag-Ärzte, Vertragsdatum: 21.08.2013, Fassung vom: 23.03.2020, Inkrafttreten: 1. Apr. 2020

[18] KBV Coronavirus: Videosprechstunden unbegrenzt möglich. https://www.kbv.de/html/1150_44943.php;. Zugegriffen: 25. Mai 2020

[19] Shenoy P, Ahmed S, Paul A (2020). Switching to teleconsultation for rheumatology in the wake of the COVID-19 pandemic: feasibility and patient response in India. Clin Rheumatol.

